# Association of neurogenic orthostatic hypotension with cognitive decline in Parkinson’s disease: a longitudinal cohort study

**DOI:** 10.3389/fneur.2026.1783953

**Published:** 2026-03-12

**Authors:** Neda Bagheri, Katherine Longardner, David Coughlin

**Affiliations:** 1 School of Medicine, University of California, San Diego, San Diego, CA, United States; 2 Department of Neuroscience, University of California, San Diego, CA, United States

**Keywords:** autonomic dysfunction, cognitive decline, longitudinal analysis, neurogenic orthostatic hypotension, Parkinson’s disease

## Abstract

**Introduction:**

Neurogenic orthostatic hypotension (nOH), a common non-motor feature of Parkinson’s disease (PD), is defined as a sustained drop in blood pressure (BP) upon standing due to autonomic dysfunction. Although prior studies support an association between nOH and cognitive impairment, its longitudinal impact on cognitive decline in PD remains insufficiently explored. We aimed to determine to what extent baseline nOH is associated with an accelerated decline in global and exploratory domain-specific cognitive functions.

**Methods:**

A retrospective longitudinal cohort study was conducted using clinical data from patients with PD evaluated at the University of California San Diego movement disorders clinics between 2012 and 2024. Participants were classified as having nOH (nOH+) or without nOH (nOH−) based on initial orthostatic BP measurements (≥20 mmHg systolic BP and/or ≥10 mmHg diastolic BP reduction within 3 minutes of standing, with a blunted heart rate response [ΔHR/ΔSBP] < 0.5 bpm/mmHg). Cognitive performance was assessed using the Montreal Cognitive Assessment (MoCA), including total scores and domain-specific subscores. Longitudinal changes in cognitive function were modeled using linear mixed-effects models, adjusting for time, nOH status, age, sex, and their interaction. Motor and non-motor symptom progression was evaluated using the MDS-UPDRS Parts I–III.

**Results:**

Patients with nOH at the first visit showed faster decline in total MoCA scores (Interaction *β* = −0.57, SE = 0.15, *p* < 0.001), reflecting declines in abstraction (Interaction *β* = −0.15, SE = 0.05, *p* < 0.01), attention (Interaction *β* = −0.13, SE = 0.04, *p* < 0.01), delayed recall (Interaction *β* = −0.18, SE = 0.06, *p* < 0.001), executive function (Interaction *β* = −0.16, SE = 0.05, *p* < 0.001), language (Interaction *β* = −0.14, SE = 0.05, *p* < 0.001), and orientation (Interaction *β* = −0.17, SE = 0.06, *p* < 0.001) subscores. No significant time × nOH interaction was observed for MDS-UPDRS Parts I-III (all *p* > 0.6), indicating comparable rates of motor and nonmotor symptom progression between nOH+ and nOH− groups.

**Discussion:**

In this single-center, real-world retrospective cohort, the presence of baseline nOH was associated with a more rapid decline in cognitive function. These findings highlight the potential importance of early autonomic assessment in PD and warrant further investigation in larger, multi-center studies to determine generalizability and to better understand the underlying mechanisms contributing to cognitive deterioration.

## Introduction

Parkinson’s disease (PD) is a progressive neurodegenerative disorder characterized by motor symptoms such as tremor, bradykinesia, rigidity, and postural instability. However, non-motor symptoms, including cognitive impairment and autonomic dysfunction, are also highly prevalent in individuals with PD and significantly contribute to morbidity and diminished quality of life (1–3). Cognitive decline in PD can vary in rate and characteristics, with commonly affected domains including executive and visuospatial function and attention, and to a lesser extent, memory, language, and orientation (4, 5).

Neurogenic orthostatic hypotension (nOH), a manifestation of autonomic failure in PD, is defined as a sustained drop in systolic blood pressure (SBP) (≥20 mmHg) and/or diastolic blood pressure (DBP) (≥10 mmHg) within 3 min of standing or head-up tilt, due to autonomic nervous system failure (6). nOH affects approximately 30% of individuals with PD (7) and may be associated with symptoms such as dizziness, fatigue, falls, and syncope (6), or may be asymptomatic.

Prior studies have reported associations between orthostatic hypotension (OH) and poorer global cognitive performance, as well as domain-specific deficits in executive function, memory, and attention (8–10). A systematic review and meta-analysis demonstrated a significant association between nOH and cognitive impairment in PD, with affected individuals exhibiting a 3.3-fold increased odds of developing mild cognitive impairment or dementia (11). However, only four of the 18 studies included in this review were longitudinal cohort studies; the rest were cross-sectional.

Given the scarcity of longitudinal studies on the relationship between nOH and cognition in PD using standardized methods, it remains unclear whether baseline nOH independently predicts the trajectory of cognitive decline in PD. This study aimed to address this gap by evaluating to what extent baseline nOH is associated with accelerated decline in global and exploratory domain-specific cognitive function in PD, and whether nOH is associated with the progression of motor and non-motor symptoms in a real-world cohort from a tertiary movement disorders clinic in the United States. Here, we expanded on our prior findings (10) by examining a larger cohort of PD patients over a longer period of time using stricter diagnostic criteria for nOH, analyzing both global and exploratory domain-specific cognitive trajectories. Based on prior work, we hypothesized that nOH would be associated with greater decline in global cognition and visuospatial and executive domains.

## Method

### Study design and setting

This retrospective longitudinal cohort study utilized data extracted from electronic medical records of patients followed at the University of California San Diego (UCSD) Movement Disorders Clinic, who provided written permission for secondary data use (UCSD Institutional Review Board protocol #111851). The dataset comprised de-identified longitudinal clinical records of PD patients evaluated between 2012 and 2024 and was downloaded in March 2024.

Demographic variables (age, sex, race/ethnicity), cognitive assessments, and motor and non-motor symptom ratings were collected across multiple outpatient visits. Autonomic function was assessed in clinic using orthostatic vital signs, including systolic and diastolic blood pressure and heart rate measured in supine and standing positions. Cognitive performance was evaluated using the Montreal Cognitive Assessment (MoCA) (12), including total scores and domain-specific subscores: abstraction, attention, delayed recall, executive function, language, and orientation. Motor and non-motor symptoms were assessed using the Movement Disorder Society–Unified Parkinson’s Disease Rating Scale (MDS-UPDRS) Parts I–III (13).

### Data collection and participants

**Figure 1:**
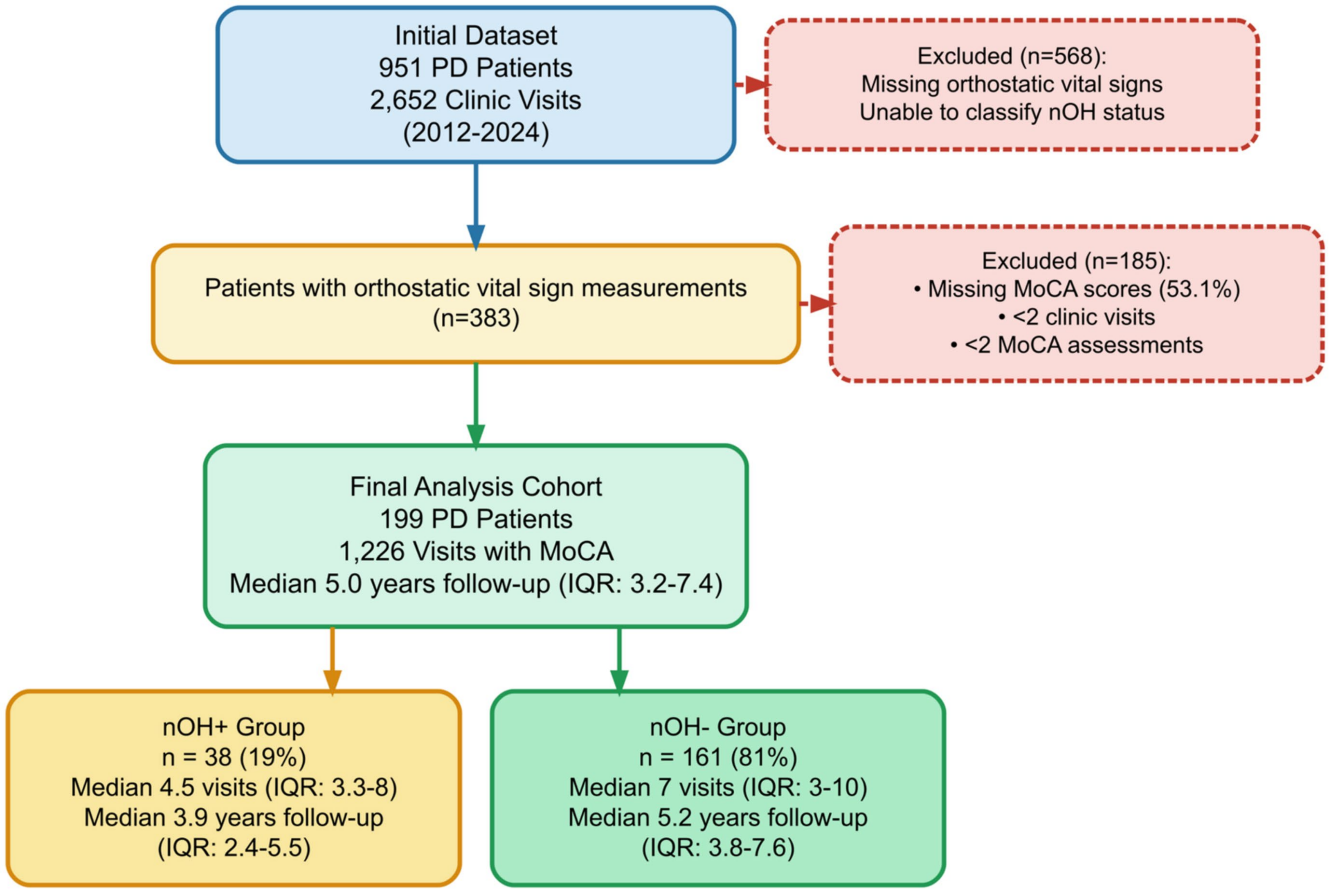
Study flow diagram. Flow diagram showing patient selection and cohort characteristics for the retrospective longitudinal study of neurogenic orthostatic hypotension and cognitive decline in Parkinson’s disease. From an initial dataset of 951 PD patients with 2,652 clinic visits collected between 2012 and 2024, participants were excluded for missing orthostatic vital signs necessary for nOH classification, missing MoCA scores (53.1% of observations), fewer than 2 clinic visits, or fewer than 2 MoCA assessments. The final analysis cohort included 199 participants with 1,226 visits, stratified by baseline nOH status using strict diagnostic criteria (≥20 mmHg systolic BP and/or ≥10 mmHg diastolic BP drop within 3 min of standing, with ΔHR/ΔSBP <0.5 bpm/mmHg). The nOH + group (*n* = 38, 19%) had a median of 4.5 visits over 3.9 years of follow-up, while the nOH- group (*n* = 161, 81%) had a median of 7 visits over 5.2 years of follow-up. Exclusion criteria are shown in dashed boxes with red borders. PD, Parkinson’s disease; nOH, neurogenic orthostatic hypotension; MoCA, Montreal Cognitive Assessment; BP, blood pressure; ΔHR/ΔSBP, change in heart rate divided by change in systolic blood pressure; IQR, interquartile range.

Eligible participants had a clinical diagnosis of PD made by a movement disorders neurologist, identified by the International Classification of Diseases 10 code G20. All participants underwent orthostatic blood pressure and heart rate assessments at their initial clinic visit (in the supine position, followed by 1 and 3 min of active standing). If patients were unable to lie flat due to limited mobility or pain, BP was measured instead in the sitting and standing positions. Participants included in the analysis had at least two outpatient visits between 2012 and 2024, and at least two recorded completed MoCA scores. Participants were excluded if they had missing orthostatic vital sign measurements necessary for nOH classification or were diagnosed with atypical parkinsonian syndromes such as multiple system atrophy or progressive supranuclear palsy. A detailed flow diagram of patient selection and exclusion criteria is provided in Figure 1.

Participants were classified as having nOH (nOH+) if they met both of the following criteria: (1) a decrease in SBP of ≥ 20 mmHg and/or DBP of ≥10 mmHg within 3 min of standing from sitting or supine (6), and (2) a ΔHR/ΔSBP ratio of <0.5 bpm/mmHg, calculated as the increase in heart rate divided by the fall in SBP from supine to standing (14). Other participants were classified as nOH − .

Cognitive performance was assessed using the MoCA which was administered in person by trained staff. The total MoCA score was selected as the primary outcome measure. Domain-specific subscores were derived by grouping individual MoCA items according to standard scoring structure (12). These domains included: visuospatial/executive function (trail-making, three-dimensional figure copying, clock drawing), naming (animal identification), attention (digit span, letter tapping, serial 7 s), language (sentence repetition, verbal fluency), abstraction (similarities), delayed recall (free recall of five words), and orientation (temporal and spatial orientation).

MDS-UPDRS Parts I, II, and III scores were extracted from clinical visits and included in the analysis when available. Higher scores on Parts I and II reflect more severe non-motor and daily living symptoms, respectively, while higher scores on Part III indicate greater motor impairment. A movement disorders neurologist administered the MDS-UPDRS Part III. There were no attempts to standardize ON/OFF status for MDS-UPDRS Part III assessments given the real-world nature of the study, and ON/OFF status was not consistently documented so could not be accounted for in the analysis.

### Statistical analysis

Baseline demographic, clinical, and autonomic variables were summarized as medians (IQR) for continuous variables and counts (percentages) for categorical variables. Group comparisons between nOH + and nOH − participants at baseline were conducted using the Wilcoxon rank-sum test for continuous variables and Fisher’s exact test for categorical variables.

Normality of continuous variables, including MoCA scores, was assessed using histograms and Q–Q plots. Due to non-normality in several variables, non-parametric tests were used for baseline comparisons. Linear mixed-effects models (LMEMs) were used to evaluate longitudinal changes in cognitive outcomes over time, as they are robust to normality violations. Residual diagnostics confirmed acceptable model fit.

Longitudinal changes in MoCA total and subdomain scores were modeled using LMEMs, with fixed effects for time, group, age, sex, and a time × group interaction, and random intercepts for participants. Missing MoCA scores were imputed within participants; no imputation was performed for MDS-UPDRS due to extensive missing data that would compromise imputation reliability.

All statistical analyses were performed using R (version 4.4.2) in RStudio (15). LMEMs were fitted with the lme4 package (16). Data handling and visualization were conducted with tidyverse (17), ggplot2 (18), and gtsummary (19).

Model diagnostics, including residual plots and Q–Q plots, confirmed that assumptions of normality, linearity, and homoscedasticity were sufficiently met for the LMEM. Residuals were approximately normally distributed, with no evidence of extreme skewness or heteroscedasticity.

## Results

### Baseline characteristics

A total of 199 participants with PD were included in the analysis, of whom 38 (19%) met criteria for nOH + on their initial assessment, and 161 (81%) did not (nOH−). Participants in the nOH + group were followed for a median of 3.9 years (IQR: 2.4–5.5) over a median of 4.5 visits (IQR: 3.3–8; range: 2–20), while the nOH − group was followed for a median of 5.2 years (IQR: 3.8–7.6) over a median of 7 visits (IQR: 3–10; range: 2–31) (*p* = 0.16). Median age was higher in the nOH + group compared to the nOH − group (75 [IQR: 68–81] vs. 71 [IQR: 66–78] years), although this difference did not reach statistical significance (*p* = 0.11). There were no differences in sex (*p* = 0.90) or primary ethnicity (*p* = 0.20) between the groups.

Autonomic measures revealed higher supine systolic and diastolic blood pressure in the nOH + group compared to the nOH − group (SBP: 142 [135–154] vs. 129 [119–138] mmHg, *p* < 0.001; DBP: 80 [73–88] vs. 74 [69–79] mmHg, *p* < 0.001). The median SBP drop was 26.5 mmHg (IQR: 18.8–35.5) in the nOH + group versus 3.0 mmHg (IQR: −5.0–12.0) in the nOH − group. The median DBP drop was 13.5 mmHg (IQR: 7.5–17.8) in the nOH + group versus −2.0 mmHg (IQR: −8.0–2.0) in the nOH − group. All differences were statistically significant (*p* < 0.001). Supine and standing heart rate did not differ between groups (*p* > 0.1 for both). The change in heart rate from supine to standing also did not differ between groups (*p* > 0.1). Table 1 summarizes baseline demographic and clinical characteristics of patients included.

**Table 1:** Baseline demographic and cognitive characteristics of Parkinson’s disease (PD) patients stratified by the presence of neurogenic orthostatic hypotension (nOH) at the initial clinical visit.

Characteristic	N	With nOH *N* = 38^1^	Without nOH *N* = 161^1^	*p*-value^2^
Age, years	199	75 (68, 81)	71 (66, 78)	0.11
Sex	199			0.9
Female		14 (37%)	57 (35%)	
Male		24 (63%)	104 (65%)	
Primary ethnicity	199			0.2
Hispanic or Latino		2 (5.3%)	10 (6.2%)	
Non-Hispanic		24 (63.2%)	72 (44.7%)	
Not Reported		1 (2.6%)	9 (5.6%)	
Unknown		11 (28.9%)	70 (43.5%)	
Primary race	199			0.9
Asian		1 (2.6%)	11 (6.8%)	
More Than One Race		2 (5.3%)	11 (6.8%)	
Other		0 (0.0%)	4 (2.5%)	
Unknown/Not Reported		4 (10.5%)	17 (10.6%)	
White		31 (81.6%)	118 (73.3%)	
SBP Supine, mmHg	199	142 (135, 154)	129 (119, 138)	<0.001
DBP Supine, mmHg	199	80 (73, 88)	74 (69, 79)	<0.001
SBP Standing 3 Min, mmHg	199	116 (104, 128)	127 (116, 136)	0.005
DBP Standing 3 Min, mmHg	199	69 (62, 76)	77 (70, 84)	<0.001
HR Supine, bpm	199	75 (63, 83)	70 (63, 79)	0.3
HR Standing 3 Min, bpm	198	77 (68, 87)	79 (69, 88)	0.4
Unknown		0	1	
Baseline Global MoCA Score	199	26.5 (25, 28)	26 (24, 28)	0.9
MDS-UPDRS Part I	24	9 (7, 11)	9 (6, 12)	>0.9
Unknown		33	142	
MDS-UPDRS Part II	22	7.5 (3, 13.5)	5.5 (4, 12)	>0.9
Unknown		34	143	
MDS-UPDRS Part III	30	25 (22, 33)	24 (19, 30)	0.6
Unknown		29	140	

1 Median (Q1, Q3); *n* (%).

2 Wilcoxon rank sum test; Fisher’s exact test.

Data are presented as medians with interquartile ranges (IQR) for continuous variables, and counts with percentages for categorical variables. Group comparisons were conducted using the Wilcoxon rank-sum test for continuous variables and Fisher’s exact test for categorical variables. Statistically significant differences were observed in supine and standing systolic blood pressure (SBP) and diastolic blood pressure (DBP), with higher supine, lower standing values in the nOH + group (all *p* < 0.01). Between-group differences in positional BP changes (supine minus 3 min standing) for both SBP and DBP are also included. PD, Parkinson’s disease; nOH, neurogenic orthostatic hypotension; SBP, systolic blood pressure; DBP, diastolic blood pressure; IQR, interquartile range, HR, heart rate, MoCA, Montreal Cognitive Assessment, MDS-UPDRS.

Baseline global cognitive function, assessed using the MoCA total score, was similar between groups at initial assessment (nOH+: 26.5 [25.0–28.0] vs. nOH−: 26.0 [24.0–28.0], *p* = 0.90). Domain-specific MoCA sub-scores, including attention, language, abstraction, and delayed recall, were also similar between groups (*p* > 0.1 for all). Baseline MDS-UPDRS Part I (*p* > 0.9), Part II (*p* > 0.9), and Part III (*p* = 0.6) scores were also similar between groups (Table 1).

### Global cognitive decline (MoCA total score)

**Figure 2:**
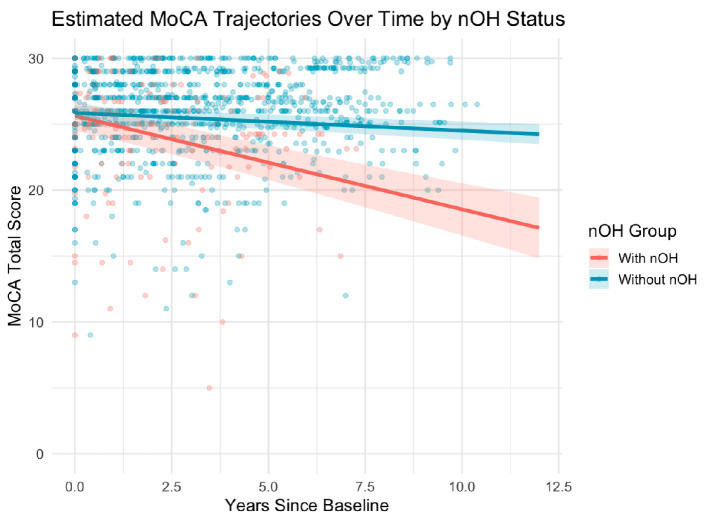
Estimated trajectories of global cognitive function over time, based on Montreal Cognitive Assessment (MoCA) total scores, stratified by baseline neurogenic orthostatic hypotension (nOH) status. Predicted values were derived from a linear mixed-effects model including fixed effects for time (years), nOH status, and their interaction, with random intercepts to account for within-subject variability. Solid lines represent model-estimated MoCA scores; shaded bands denote 95% confidence intervals, and semi-transparent dots indicate individual observations. Participants with baseline nOH (nOH+) exhibited significantly faster cognitive decline over time compared to those without nOH (nOH−), as indicated by the significant Time × nOH interaction (*p* < 0.001). MoCA, Montreal Cognitive Assessment; nOH, neurogenic orthostatic hypotension.

MoCA scores were available for all 1,226 visits (184 visits for nOH+, 1,042 visits for nOH−) across the 199 participants included in the analysis. Linear mixed-effects modeling revealed a greater rate of global cognitive decline over time in nOH + patients compared to nOH−, accounting for varying follow-up durations and visit intervals across individuals. The overall rate of MoCA decline in the nOH + group was −0.71 points per year (95% CI: −0.90 to −0.52, *p* < 0.001), observed over a median follow-up period of 5.0 years (IQR: 3.2–7.4 years). In contrast, nOH − participants experienced a slower rate of decline at −0.13 points per year (95% CI: −0.24 to −0.01, *p* = 0.04). The between-group difference in annual decline rate was 0.57 points per year (95% CI: 0.38 to 0.77, *p* < 0.001), with more rapid global cognitive deterioration in the nOH + group (Figure 2).

### Exploratory analysis: domain-specific MoCA sub-scores

**Figure 3:**
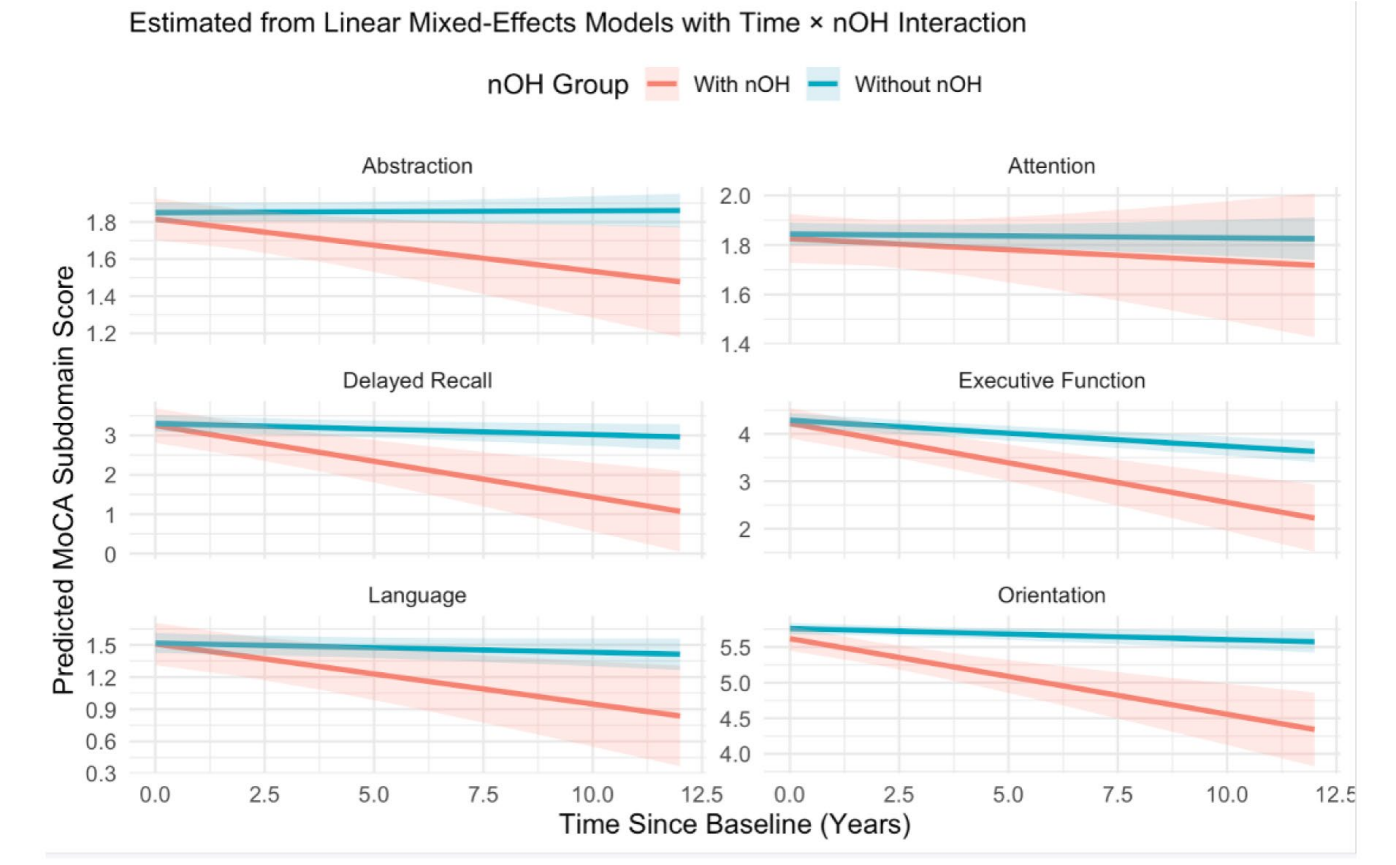
Predicted trajectories of domain-specific cognitive function over time in Parkinson’s disease patients with and without baseline neurogenic orthostatic hypotension (nOH), based on separate linear mixed-effects models for each MoCA subdomain. Panels display estimated changes in Abstraction, Attention, Delayed Recall, Executive Function, Language, and Orientation scores. The x-axis indicates years since baseline; the y-axis shows model-predicted subdomain scores. Red and blue lines represent the nOH + and nOH − groups, respectively, with shaded bands denoting 95% confidence intervals. Participants with nOH exhibited significantly steeper declines across multiple cognitive domains, as reflected by significant Time × nOH interactions. All models included fixed effects for time, group, and their interaction, with random intercepts for participants. MoCA, Montreal Cognitive Assessment; nOH, neurogenic orthostatic hypotension.

In exploratory analyses, participants with baseline nOH exhibited faster decline across multiple cognitive domains compared to those without baseline nOH, as shown in Figure 3. Significant Time × nOH status interactions were found for memory (*β* = 0.15, 95% CI: 0.10 to 0.21, *p* < 0.001), executive function (*β* = 0.11, 95% CI: 0.06 to 0.16, *p* < 0.001), attention (β = 0.09, 95% CI: 0.04 to 0.14, *p* = 0.001), language (*β* = 0.08, 95% CI: 0.03 to 0.13, *p* = 0.002), and orientation (*β* = 0.09, 95% CI: 0.04 to 0.14, *p* < 0.001). A modest but statistically significant interaction was also observed for abstraction (*β* = 0.03, 95% CI: 0.002 to 0.06, *p* = 0.036). These findings indicate that baseline nOH is associated with accelerated decline in exploratory domain-specific cognitive functions in PD (Supplementary Table 1; Table 2).

**Table 2:** Results from a linear mixed-effects model evaluating longitudinal changes in global cognitive function, measured by the Montreal Cognitive Assessment (MoCA) total score, in Parkinson’s disease (PD) patients stratified by baseline neurogenic orthostatic hypotension (nOH) status.

Characteristic	Estimate (β)	SE	95% CI (Lower)	95% CI (Upper)	*p*-value
Baseline MoCA (nOH+)	25.63	0.57	24.50	26.76	<0.001
MoCA Change per Year (nOH+)	−0.71	0.10	−0.90	−0.52	<0.001
Baseline MoCA Difference (Between nOH + & nOH-)	0.23	0.64	−1.03	1.48	0.723
Difference in MoCA Decline Rate per Year (Between nOH + & nOH-)	0.57	0.10	0.38	0.77	<0.001

Baseline MoCA scores and annual rates of decline are presented for the nOH + group, with contrasts indicating differences in the rate of decline for the nOH − group. A significant interaction between time and nOH status reflects faster cognitive deterioration in the nOH + group. Random intercepts were included to account for within-subject variability. Model: MoCA Total Score ~ Time_Years * nOH Group + (1 | Code). PD, Parkinson’s disease; nOH, neurogenic orthostatic hypotension; MoCA, Montreal Cognitive Assessment.

### Longitudinal motor and non-motor symptom progression (MDS-UPDRS parts I–III)

MDS-UPDRS parts I, II, and III scores were available over an average follow-up period of 4.5 years (SD: 2.6). Fixed effects for time, nOH status, age, sex, and their interactions are summarized in Tables 3a–c.

**Table 3:** Longitudinal analysis of motor and non-motor symptom progression (MDS-UPDRS Parts I–III) by neurogenic orthostatic hypotension status.

Characteristic	Beta	95% CI	*p*-value
3a. Part I: non-motor symptoms (MDS-UPDRS I)			
(Intercept)	0.71	−11, 13	>0.9
Time Years	0.71	−0.30, 1.7	0.2
nOH Group			
With nOH	—	—	
Without nOH	0.29	−4.2, 4.8	0.9
Age	0.14	−0.02, 0.30	0.10
Sex			
Female	—	—	
Male	−3.6	−6.5, −0.84	0.012
Time Years * nOH Group			
Time Years * Without nOH	−0.25	−1.4, 0.85	0.7
3b. Part II: motor activities of daily living (MDS-UPDRS II)			
(Intercept)	−11	−24, 1.3	0.080
Time Years	0.84	−0.12, 1.8	0.086
nOH Group			
With nOH	—	—	
Without nOH	−1.6	−6.3, 3.1	0.5
Age	0.27	0.10, 0.43	0.002
Sex			
Female	—	—	
Male	0.03	−2.9, 3.0	>0.9
Time Years * nOH Group			
Time Years * Without nOH	−0.31	−1.4, 0.74	0.6
3c. Part III: motor examination (MDS-UPDRS III)			
(Intercept)	1.3	−15, 17	0.9
Time Years	0.61	−1.1, 2.3	0.5
nOH Group			
With nOH	—	—	
Without nOH	−5.3	−12, 1.5	0.13
Age	0.35	0.14, 0.56	0.001
Sex			
Female	—	—	
Male	1.9	−2.0, 5.9	0.3
Time Years * nOH Group			
Time Years * Without nOH	−0.05	−1.9, 1.8	>0.9

Fixed effects estimate from linear mixed-effects models evaluating longitudinal changes in MDS-UPDRS Parts I (nonmotor symptoms), II (motor experiences of daily living), and III (motor examination) in relation to baseline neurogenic orthostatic hypotension (nOH) status. Each model included fixed effects for time (years from baseline), nOH group, age, sex, and their interactions, with random intercepts to account for within-subject variability. No significant Time × nOH interactions were observed, indicating similar rates of motor and nonmotor symptom progression between nOH + and nOH − groups. Age was significantly associated with worsening scores in Parts II and III, and male sex was associated with lower symptom burden in Part I. CI, Confidence Interval. MDS-UPDRS, Movement Disorder Society–Unified Parkinson’s Disease Rating Scale; nOH, neurogenic orthostatic hypotension.

For MDS-UPDRS Part I (Non-Motor Activities of Daily Living), there was no interaction between time and nOH status (*β* = −0.25, 95% CI: −1.4 to 0.85, *p* = 0.7), indicating similar nonmotor symptom progression across groups. The effect of time was also non-significant (*β* = 0.71, 95% CI: −0.30 to 1.7, *p* = 0.2). However, male sex was significantly associated with lower burden of nonmotor activities of daily living scores than women (*β* = −3.6, 95% CI: −6.5 to −0.84, *p* = 0.012).

For MDS-UPDRS Part II (Motor Experiences of Daily Living), the interaction between time and nOH status was not significant (*β* = −0.31, 95% CI: −1.4 to 0.74, *p* = 0.60). The main effect of time did not reach statistical significance (*β* = 0.84, 95% CI: −0.12 to 1.8, *p* = 0.086). Older age was associated with higher MDS-UPDRS Part II scores (*β* = 0.27, 95% CI: 0.10 to 0.43, *p* = 0.002).

For MDS-UPDRS Part III (Motor Examination), motor examination scores did not change over time (*β* = 0.61, 95% CI: −1.1 to 2.3, *p* = 0.5). The rate of motor symptom progression did not differ between nOH + and nOH − groups (Time × nOH interaction: *β* = −0.05, 95% CI: −1.9 to 1.8, *p* > 0.9). Older age was associated with worsening motor examination scores (*β* = 0.35, 95% CI: 0.14 to 0.56, *p* = 0.001).

In summary, while global cognition and multiple cognitive subdomains deteriorated more rapidly in participants with nOH, the progression of motor and non-motor symptoms, as measured by MDS-UPDRS Parts I–III, did not significantly differ between PD patients with nOH + at baseline and those without over time (Table 4).

**Table 4:** Results from linear mixed-effects models evaluating the association between baseline neurogenic orthostatic hypotension (nOH) and longitudinal trajectories of domain-specific cognitive function in Parkinson’s disease (PD).

Domain	Baseline (nOH+)	Δ Baseline (nOH − vs. nOH+)	*p*-value (Baseline)	Δ Slope (Time × nOH)	*p*-value (Slope)
Abstraction	1.82	0.03	0.582	0.03	0.036
Attention	5.5	0.02	0.913	0.09	0.001
Delayed Recall	3.25	0.05	0.831	0.15	< 0.001
Executive	4.22	0.06	0.728	0.11	< 0.001
Language	2.3	0.02	0.863	0.08	0.002
Orientation	5.62	0.14	0.14	0.09	< 0.001

Longitudinal effects of baseline neurogenic orthostatic hypotension on MoCA cognitive subdomains. Models included fixed effects for time (years), nOH status, and their interaction, with random intercepts to account for within-subject variability. The table reports baseline subdomain scores for the nOH + group, baseline differences between groups, and group differences in annual rates of change (Time × nOH interaction). Significant interactions reflect accelerated decline in specific cognitive domains among participants with baseline nOH. PD, Parkinson’s disease; nOH, neurogenic orthostatic hypotension.

### Sensitivity analyses

To assess the robustness of the primary findings, several sensitivity analyses were conducted. First, the linear mixed-effects model (LMEM) evaluating global cognitive decline by MoCA score was re-estimated with additional covariates for age and sex. The results remained consistent with the unadjusted model: the interaction between time and baseline nOH status remained highly significant (*β* = +0.56, *p* < 001), confirming that participants without nOH demonstrated slower cognitive decline over time. Age was also a significant predictor of lower MoCA scores (*β* = −0.10, *p* < 0.001), while sex was not (*p* = 0.30; Table 5).

**Table 5:** Adjusted linear mixed-effects model of MoCA score decline by baseline nOH status.

Predictor	Beta	95% CI	*p*-value
(Intercept)	33	28, 37	<0.001
Time (years)	−0.70	−0.89, −0.51	<0.001
Group (ref: With nOH)			
nOH-	—	—	
nOH+	0.11	−1.3, 1.5	0.9
Age (years)	−0.10	−0.15, −0.05	<0.001
Sex (ref: Female)			
Female	—	—	
Male	0.48	−0.53, 1.5	0.3
Time × Group Interaction			
Time (years) * nOH+	0.56	0.36, 0.76	<0.001

Fixed effects estimate from a linear mixed-effects model evaluating longitudinal changes in Montreal Cognitive Assessment (MoCA) total scores based on baseline neurogenic orthostatic hypotension (nOH) status. The model adjusts for time (years from baseline), age, and sex, and includes random intercepts for within-subject variability. A significant interaction between time and nOH status indicates faster cognitive decline among participants with baseline nOH. The nOH − group served as the reference category in all models. CI, Confidence Interval; MoCA, Montreal Cognitive Assessment; nOH, neurogenic orthostatic hypotension.

### Exploratory analysis: supine hypertension and cognitive decline

An exploratory analysis examined whether supine hypertension (SH, defined as supine SBP ≥ 140 mmHg or DBP ≥ 90 mmHg) (20), modified the association between nOH and cognitive decline. Among the 38 nOH + participants, 21 (55.3%) had concurrent SH. Linear mixed-effects modeling revealed that participants with both nOH and SH (nOH+/SH+) experienced the fastest cognitive decline (−0.88 points/year, 95% CI: −1.17 to −0.58, *p* < 0.001), while those with nOH but without SH (nOH+/SH−) showed intermediate decline (−0.39 points/year, 95% CI: −0.74 to −0.05, *p* = 0.082). Participants without nOH (nOH−) showed minimal decline (−0.13 points/year, 95% CI: −0.19 to −0.08, *p* < 0.001) (Supplementary Table 1).

## Discussion

This single-center retrospective study provides evidence that nOH is associated with an accelerated decline in global and exploratory domain-specific cognitive function in individuals with PD. Although baseline total and subdomain MoCA scores were similar between nOH + and nOH − groups, participants with nOH demonstrated a faster longitudinal deterioration in global cognition, specifically in memory, executive function, language, and orientation tasks. These findings support the hypothesis that cardiovascular autonomic dysfunction, specifically nOH and accompanying SH, are associated with cognitive deterioration in PD and highlight the potential value of early autonomic assessment in identifying patients at higher risk of cognitive decline. Sensitivity analyses further support the validity and stability of these findings, demonstrating that the observed association between baseline nOH and accelerated cognitive decline in PD is not confounded by demographic factors.

The association between nOH and cognitive decline in PD observed in this study aligns with prior research a strong association between cardiovascular autonomic dysfunction and cognitive impairment in PD. Using an earlier version of the current dataset (through 2019), we previously found that individuals with PD and OH experienced greater global cognitive decline over time (10). Importantly, the current study incorporates extended follow-up data through 2024, including a larger cohort of 199 participants (compared to 42 in the prior analysis), offering a more comprehensive perspective and incorporating exploratory cognitive domain-specific analyses. Our findings extend previous observations by providing longitudinal evidence that nOH affects multiple specific cognitive domains without associated motor worsening, highlighting the need for further investigation into the underlying pathophysiological pathways.

It remains unclear whether the relationship between nOH and cognitive impairment is causative or merely associative (21). It has been hypothesized that repeated episodes of cerebral hypoperfusion resulting from nOH may contribute directly to neurodegenerative and/or chronic cerebrovascular changes in PD, with prior studies showing that OH is associated with white matter hyperintensities (22) and temporal cortex atrophy on brain MRI (23). Alternatively, emerging cluster analyses suggest that patients with both nOH and cognitive impairment may represent a biologically distinct “diffuse malignant” subtype of PD, characterized by widespread autonomic and cognitive symptoms and more rapid progression (24–26). This raises the possibility that autonomic dysfunction and cognitive decline may co-occur as manifestations of a more aggressive PD phenotype, rather than arising through a direct causal mechanism.

However, our results showed no significant difference in motor and non-motor symptom progression as measured by MDS-UPDRS Parts I-III between groups, although the relative missingness of UPDRS data may have limited our ability to detect subtle differences. These findings raise the possibility that nOH-related cognitive decline may occur through cerebrovascular mechanisms (chronic cerebral hypoperfusion and/or related supine hypertension) that are at least partially independent of motor symptom progression, though further investigation with more complete motor assessment data is needed to confirm this observation.

Notably, our exploratory analysis revealed a graded relationship between nOH, SH, and the severity of cognitive decline, with the fastest decline observed in participants with both nOH and SH (nOH+/SH+), intermediate decline in those with nOH alone (nOH+/SH−), and minimal decline in those without nOH. This gradient suggests that the severity of blood pressure dysregulation may be a key contributing factor to cognitive deterioration in PD, rather than nOH simply representing a marker of a more aggressive disease phenotype. SH is a common manifestation of cardiovascular dysautonomia in PD and is associated with increased morbidity and mortality (27, 28). Our findings suggest that the combination of nOH and SH may confer additional risk for accelerated cognitive decline, warranting further investigation.

The association between nOH and cognitive decline emphasizes the importance of autonomic function assessment in the clinical management of PD. Early identification of nOH may assist in recognizing individuals at increased risk for accelerated cognitive deterioration, enabling more tailored monitoring strategies.

Additionally, numerous interventions exist to treat OH, which are feasible strategies in PD. Non-pharmacological methods to raise blood pressure include increasing salt and fluid intake, wearing compression garments, and utilizing physical counter-maneuvers (6, 28, 29). Furthermore, various oral medications, including midodrine, droxidopa, and fludrocortisone, exist to treat symptoms of OH (6), although further interventional longitudinal studies are necessary to determine whether treating nOH can improve cognitive outcomes in PD.

### Limitations

Several limitations should be noted. This retrospective study design using real-world clinical data, this design has several inherent limitations. Selection bias may have occurred, as patients with missing data, more severe disease, or irregular follow-up patterns were excluded from analysis. In the original dataset of 951 patients with 2,652 clinic visits, MoCA scores were collected at irregular intervals based on clinical follow-up and were missing for 53.1% of observations. Participants with missing MoCA scores, missing nOH classification at baseline, or insufficient follow-up data (<2 visits) were excluded, reducing the sample to 199 patients with 1,226 visits. While this approach ensured complete MoCA data for analysis (Supplementary Table 2), it may have excluded patients with more severe disease or irregular follow-up patterns, potentially limiting generalizability. Our relatively small sample sizes for nOH + participants may limit statistical power.

MDS-UPDRS Parts I, II, and III subscores were not systematically collected during routine clinical visits and missingness of data may contribute to findings noted (Supplementary Table 2). The missingness of MDS-UPDRS data (17.0–28.8% availability) compared to complete MoCA data availability (100% by design due to inclusion criteria) may have limited our statistical power to detect associations between nOH and motor symptom progression. While we found no significant difference in motor or non-motor symptom progression by UPDRS between groups, this finding should be interpreted cautiously given the incomplete assessment schedule and potential for Type II error. Additionally, the observational nature of the study precludes causal inference regarding the relationship between nOH and cognitive decline. The absence of detailed clinical data, including levodopa dose [which may lower blood pressure (30, 31)], disease duration and stage, and medical comorbidities, limited the ability to adjust for these potential confounders. Detailed medication data, including treatments specifically for nOH (e.g., midodrine, droxidopa, fludrocortisone) were not systematically collected in this retrospective study. This limits our ability to assess whether pharmacological management of nOH influences cognitive outcomes or whether the observed associations are modified by treatment status. Prospective studies with standardized assessment protocols, comprehensive covariate collection, and longer follow-up would be needed to address these limitations and confirm these findings.

Ultimately, these limitations are reflections of the study design, which used real-world data collected in a clinical setting. Furthermore, this is a single centered study in the United States with a largely Caucasian population, which may also limit generalizability. Additionally, while the MoCA is a widely used cognitive screening tool in PD (32), it has inherent limitations. The MoCA was designed as a brief global screening instrument for rapid clinical assessment rather than comprehensive neuropsychological evaluation and may have limited sensitivity for detecting subtle domain-specific changes, particularly in executive function and visuospatial domains, and for measuring longitudinal decline over several years (33, 34). Furthermore, the MoCA subsections were not originally designed or validated for independent domain-specific assessment, which may affect the precision of subdomain trajectory estimates in this study (30). A comprehensive neuropsychological battery, which would include multiple validated tests per cognitive domain and more extensive evaluation of executive function, memory, attention, and visuospatial abilities, would provide more detailed and sensitive assessment of domain-specific cognitive changes (33). However, such testing was not routinely performed in this real-world clinical cohort due to time and resource constraints typical of outpatient clinics. Despite these limitations, the MoCA remains a practical tool for longitudinal cognitive assessment in PD clinical settings.

This study used a conservative approach to classify nOH, requiring both a sustained orthostatic blood pressure drop and a blunted heart rate response (6, 14). While this strategy reduced potential misclassification, it may have excluded individuals with non-neurogenic OH who still experience clinical effects of autonomic dysfunction. This study classified nOH status based on orthostatic vital signs obtained at the initial clinic visit only. We did not systematically reassess nOH status at subsequent visits, as orthostatic blood pressure measurements were inconsistently available at follow-up visits in this real-world clinical cohort. Therefore, we cannot account for incident nOH (conversion from nOH- to nOH+) that may have occurred during the follow-up period. Prior work by Longardner et al. (10) in a smaller cohort found that approximately 70% of PD patients developed OH over a median follow-up of 3.5 years, suggesting that incident nOH is common in this population. If patients in the nOH- group of the present study developed nOH during follow-up and subsequently experienced accelerated cognitive decline, this misclassification would bias our results toward the null (i.e., underestimate the true association between nOH and cognitive decline). Future prospective studies with systematic repeated assessment of autonomic function are needed to better characterize the timing of nOH onset and its relationship to cognitive decline trajectory. Furthermore, follow-up duration was calculated based on time from the first clinic visit rather than estimated disease or symptom onset, which may limit precision in assessing the true trajectory of cognitive decline. Although linear mixed-effects models were used to adjust for varying time intervals and number of follow-up visits, the heterogeneity in individual follow-up schedules remains a potential limitation.

## Conclusion and future directions

This retrospective longitudinal cohort study supports that baseline nOH and SH are associated with a more rapid decline in global cognition and several exploratory domain-specific cognitive functions in PD. However, further research is necessary to confirm these results in larger, multi-center cohorts with standardized assessment protocols and to determine the pathophysiological mechanisms of the relationship between nOH to cognitive decline.

Future studies should explore whether targeted interventions for cardiovascular autonomic dysfunction in PD, both pharmacologic and non-pharmacologic, can mitigate cognitive decline. Specifically, comparing cognitive trajectories between treated and untreated nOH patients would help determine whether nOH management has neuroprotective benefits and could inform clinical decision-making regarding early initiation of nOH treatment in PD. Prospective studies with repeated systematic assessment of autonomic function are needed to determine whether incident nOH (new-onset nOH during follow-up) carries the same prognostic significance as baseline nOH, and to identify the critical window during which nOH onset most strongly influences cognitive decline. Additionally, mechanistic investigations using neuroimaging studies could help elucidate the role of cerebral hemodynamic changes in cognitive deterioration associated with nOH. Prospective research incorporating comprehensive clinical and biomarker data will be critical for advancing understanding of this association and guiding therapeutic strategies.

## Data Availability

The datasets presented in this article are not readily available due to institutional policy and patient privacy agreements, the de-identified clinical data cannot be shared publicly or made available upon request. Requests to access the datasets should be directed to NB, neda.bagheri62@gmail.com.
