# Supplementary material for: Association of neurogenic orthostatic hypotension with cognitive decline in Parkinson’s disease: a longitudinal cohort study
Source: Front Neurol. 2026 Mar 12;17:1783953. doi: 10.3389/fneur.2026.1783953 (PMC13020547; doi:10.3389/fneur.2026.1783953)
Supplement: Supplementary file 1 [file Table_1.docx]

| **Supplementary Table 1. Association Between Supine Hypertension, Neurogenic Orthostatic Hypotension, and Rate of Cognitive Decline** | | | |
| --- | --- | --- | --- |
| **Group** | **N** | **Annual MoCA Decline Rate (95% CI)** | **P-value** |
| nOH−/SH− | 161 | −0.13 (−0.19, −0.08) | <0.001 |
| nOH+/SH− | 17 | −0.39 (−0.74, −0.05) | 0.082 |
| nOH+/SH+ | 21 | −0.88 (−1.17, −0.58) | <0.001 |
| Linear mixed-effects model with random intercepts for participants. Supine hypertension (SH) is defined as supine systolic blood pressure ≥140 mmHg or diastolic blood pressure ≥90 mmHg. | | | |
| CI = confidence interval; MoCA = Montreal Cognitive Assessment; nOH = neurogenic orthostatic hypotension; SH = supine hypertension. | | | |
| Data from 199 participants with Parkinson's disease across 1226 clinic visits. | | | |
