# Supplementary material for: Association of neurogenic orthostatic hypotension with cognitive decline in Parkinson’s disease: a longitudinal cohort study
Source: Front Neurol. 2026 Mar 12;17:1783953. doi: 10.3389/fneur.2026.1783953 (PMC13020547; doi:10.3389/fneur.2026.1783953)
Supplement: Supplementary file 2 [file Table_2.docx]

| **Supplementary Table 2. Data Availability for Cognitive and Motor Assessments** | | | | |
| --- | --- | --- | --- | --- |
| **Assessment** | **Available**  **n** | **Missing**  **n** | **Total Visits**  **n** | **Available**  **%** |
| Montreal Cognitive Assessment (MoCA) | 1,226 | 0 | 1,226 | 100.0 |
| MDS-UPDRS Part I (Non-Motor Symptoms) | 209 | 1,017 | 1,226 | 17.0 |
| MDS-UPDRS Part II (Motor Activities of Daily Living) | 212 | 1,014 | 1,226 | 17.3 |
| MDS-UPDRS Part III (Motor Examination) | 353 | 873 | 1,226 | 28.8 |
| MDS-UPDRS = Movement Disorder Society-Unified Parkinson's Disease Rating Scale; MoCA = Montreal Cognitive Assessment. | | | | |
| Data represent the number of clinic visits with complete assessment data out of 1226 total visits from 199 participants with Parkinson's disease. | | | | |
